# Our Defence against Smallpox

**Published:** 1920-05-22

**Authors:** 


### OUR DEFENCE AGAINST SMALLPOX.

The main facts brought to practitioners' notice in the following publication emanating from the Scottish Board of Health are not perhaps remarkable for their novelty, but they give us at least a correct view of the smallpox situation in Glasgow, and the advice provided is of such value that it is difficult too often to repeat it.

The Glasgow Outbreak.

An outbreak of smallpox has occurred in Glasgow district and threatens to become epidemic. The first case sickened towards the end of February, and since then seventy-eight cases have been notified. Outside of Glasgow eleven cases have already come under the notice of the Public Health, of which nine occurred in the West and two in the East of Scotland. The type of disease is severe, with marked fatality among the unvaccinated, and, it may be assumed, with infectivity broadly corresponding to the severity. It will be realised that there is reason to fear a wide dissemination of the disease owing to the considerable passenger traffic between Glasgow and other parts of the country, which will be greatly enhanced during the coming holiday months. The fact that a high percentage of the population under thirteen years of age is unvaccinated—it was in 1907 that the conscientious-objection clause was incorporated in the Vaccination Acts—is also a matter for grave anxiety.

The Scottish Board of Health accordingly desire to impress upon medical practitioners the need for close co-operation with the Public Health in their efforts to prevent the spread of the disease, and with a view to securing early diagnosis of any suspected case which may come under their notice. In cases of doubt it would appear to be advisable to communicate with the Medical Officer of Health of the area at the earliest moment, in order that, should suspicion be confirmed, all necessary precautions may be taken pending removal to hospital. Where no doubt exists the patient and all immediate "contacts" should be warned to remain *where seen* until the arrival of the sanitary officials. The danger to others of infected persons travelling by car or other public vehicle should be kept in view and impressed on patients.

In the matter of general preventive measures, the possible danger of smallpox becoming epidemic can be very greatly mitigated by a general resort on the part of the public to vaccination and re-vaccination. Experience, however, shows that the protection which successful vaccination affords is not ordinarily sought until a wholesale dread of contracting smallpox has been instilled by actual proximity of the disease and the occurrence of deaths therefrom. In this connection the Board feel that invaluable assistance can be rendered by the general practitioner, not only in vaccinating those who desire to be vaccinated, but by persuading all members of his *clientèle* —and especially the parents or guardians of unvaccinated children—to have the operation performed without delay. The number of unvaccinated children is known to be very high. The disease shows marked virulence in a considerable proportion of the cases. There is grave danger of an epidemic. The primary preventive measure is vaccination. It is unnecessary to point out to practitioners the disastrous results which will arise from failure on the part of the population to avail themselves of the only effective means of warding off or mitigating the effects of the disease.

Particularly would we draw attention to this need for more widespread vaccination of infants and urge that the public should repeatedly be shown the value of such measures.

